# Oxidative stress increases Fas ligand expression in endothelial cells

**DOI:** 10.1186/1476-9255-3-11

**Published:** 2006-07-19

**Authors:** Mayumi Suzuki, Kazutetsu Aoshiba, Atsushi Nagai

**Affiliations:** 1 First Department of Medicine, Tokyo Women's Medical University 8-1 Kawada-cho, Shinjuku-ku, Tokyo 162-8666, Japan

## Abstract

**Background:**

Fas ligand (FasL) induces apoptosis in Fas-bearing target cells, such as leukocytes, and up-regulation of FasL expression on the endothelium may contribute to anti-inflammatory reactions that attenuate leukocyte extravasation during inflammation. Since oxidants generated during inflammation and cigarette smoking may modulate endothelial function, we examined the effect of H_2_O_2 _and cigarette smoke on endothelial FasL expression.

**Methods:**

Human umbilical vein endothelial cells (HUVECs) were exposed to nontoxic concentrations of H_2_O_2 _and cigarette smoke extracts (CSE). Membrane FasL expression was assessed by immunostaining with anti-FasL antibody followed by either monolayer-cell-based spectrofluorimetry or flow cytometry. Soluble FasL in culture supernatants was measured by enzyme-linked immunosorbent assay. For the cytotoxic assay, HUVECs were exposed to H_2_O_2 _and co-cultured with neutrophils. Neutrophils were stained by a peroxidase/diaminobenzidine-based reaction, and apoptosis was evaluated on the basis of nuclear morphology after Giemsa staining. To analyze in vitro FasL expression in arteries, rat thoracic aortas were incubated with H_2_O_2_, and paraffin-embedded sections were prepared for immunohistochemistry with anti-FasL antibody.

**Results:**

Exposure of HUVECs to H_2_O_2 _dose-dependently increased their levels of both membrane and soluble forms of FasL expression. CSE exposure also caused increased levels of FasL expression, but the increase was partially inhibited by the addition of catalase. When co-cultured with neutrophils, HUVECs exposed to H_2_O_2 _significantly promoted neutrophil apoptosis. Rat thoracic aortas incubated with H_2_O_2 _exhibited increased FasL expression on their endothelium.

**Conclusion:**

Low levels of oxidative stress increase FasL expression on endothelial cells, thereby potentially reducing leukocyte extravasation and tissue damage.

## Background

Fas ligand (FasL) is a type II membrane protein belonging to the tumor necrosis factor (TNF) family that induces apoptosis in target cells bearing the receptor Fas [1]. The role of the Fas-FasL system has been best characterized in the immune system. Interactions between Fas and FasL are functionally involved in tissue-specific regulation of various immune processes. For example, FasL expression has been detected in immune-privileged tissues, such as the eye and testis, which are protected from destructive immune responses by inducing apoptosis of infiltrating Fas-bearing immune cells [2]. In this way FasL expression in some tissues contributes to their immune-privileged status.

Endothelial cells express Fas but are normally resistant to Fas-mediated apoptosis [3,4]. On the other hand, recent studies have shown that endothelial cells express FasL constitutively and therefore regulate leukocyte extravasation [5]. Administration of the proinflammatory cytokine TNFα down-regulates FasL expression on endothelial cells and promotes inflammation, whereas over-expression of FasL on the endothelium attenuates leukocyte extravasation [5]. FasL over-expression on the endothelium of arteries also inhibits intimal hyperplasia and ischemia-reperfusion injury in animals [6,7]. Thus, up-regulation of FasL expression on the endothelium may contribute to anti-inflammatory reactions by reducing leukocyte transmigration in tissue.

Oxidants are potential mediators involved in the regulation of FasL expression. Oxidants are highly reactive compounds generated during normal metabolism as well as in various pathological states, such as inflammation, ischemia/reperfusion injury, hyperoxia, and radiation injury. Previous studies have shown that increased oxidative stress induces FasL expression by T-lymphocytes [8], microglial cells [9], and intestinal epithelial cells [10], suggesting that oxidative stress is involved in the FasL-mediated apoptotic mechanism of Fas-bearing target cells. However, little information exists about the role of oxidative stress in FasL expression by endothelial cells. Since endothelial cells are targets of the oxidative stress generated by inflammatory cells, endothelial cells themselves, and exogenous pro-oxidants, such as hyperoxia and cigarette smoke [11], we investigated whether oxidative stress up-regulates FasL expression in endothelial cells.

## Methods

### Preparation of the cigarette smoke extracts (CSE)

Aqueous CS extracts (CSE) were prepared by the method described previously, with a modification [12]. Immediately before use, mainstream smoke was generated with one cigarette (Peace^®^; Japan Tobacco Inc.) by drawing consecutive puffs into a 20-ml plastic syringe with a stopcock connected through one port to a glass vessel containing 3 ml of phosphate-buffered saline (PBS). A 20-ml puff drawn over 1 second was obtained at 10-second intervals. Each puff was held for 3 seconds and bubbled through the PBS for 5 seconds. One cigarette yielded an average of 45 puffs by this procedure. The aqueous CSE was diluted in culture medium before use, and the CSE solutions were prepared by the same person (M.S.) by exactly the same method, and they were used within 5 minutes after preparation.

### Cell culture

Primary human umbilical vein endothelial cells (HUVECs) were obtained from Clonetics (San Diego, CA). Frozen cells were thawed, plated onto a 100-mm dish (Falcon^® ^Becton Dickinson Labware, Franklin Lakes, NJ) precoated with bovine type I collagen (Vitrogen^®^; Cohesion Technologies, Palo Alto, CA), and grown in endothelial growth medium (EBM-2 containing hydrocortisone, hFGF-B, VEGF, R3-IGF-1, ascorbic acid, heparin, 2% FBS, hEGF, gentamicin, and amphotericin B; Clonetics CC-4176). When the cells reached 70% confluence, they were trypsinized with 0.1% trypsin (Gibco BRL^® ^Life Technologies, Inc., Grand Island, NY), and 2% EDTA (Gibco BRL^®^), neutralized with soybean trypsin inhibitor (Gibco BRL^®^), rinsed once with PBS, and seeded onto a new dish. Cells between passages 3 and 5 were used in the experiments. Human neutrophils from EDTA-anticoagulated venous blood specimens obtained from healthy volunteers were isolated by dextran sedimentation and centrifugation on a Histopaque gradient (without endotoxin; Sigma-Aldrich Japan, Tokyo, Japan), as previously described [12]. The purity of the neutrophil populations was >95% based on May-Grunewald-Giemsa staining, and neutrophil viability determined by trypan blue dye exclusion was >98%.

### Cell treatment

HUVECs at a density of (10^4 ^cells/mm^2^) were plated onto 96-well plates (Falcon^®^), 8-well chamber slides (Lab-Tek Nalge^® ^Nunc International, Tokyo, Japan), or onto 60-mm plates (Falcon^®^) precoated with type I collagen, and grown in endothelial growth medium. When the cells reached confluence, they were incubated for 8 hours with EBM-2 medium lacking growth factors, and then exposed to different concentrations of H_2_O_2 _(Wako Pure Chemical Industries, Ltd., Osaka, Japan) or CSE for 16 hours in the presence or absence of catalase (500 U/ml; Sigma-Aldrich). Cell viability after exposure to H_2_O_2 _or CSE was assessed by staining with 40 μg/ml of propidium iodide (Sigma-Aldrich) and spectrofluorimetry at an excitation wavelength of 530 nm and emission wavelength of 590 nm, as described previously [13].

### Analysis of FasL expression

Membrane FasL expression in HUVECs was analyzed by cell-monolayer-based spectrofluorimetry or flow cytometry. For cell monolayer-based spectrofluorimetry, HUVECs cultured on 96-well plates (Falcon^®^) were fixed with cold 1% paraformaldehyde for 15 min. After rinsing with PBS, plates were blocked with blocking buffer (PBS containing 3% bovine serum albumin and 2% goat serum: Sigma-Aldrich) and incubated for 60 minutes at room temperature with monoclonal mouse anti-FasL antibody (NOK1, 8 μg/ml; Pharmingen, San Diego, CA) or isotype-matched mouse nonimmune IgG in blocking buffer. The plates were rinsed 3 times with PBS and incubated with FITC-conjugated anti-mouse IgG antibody (5 μg/ml; Southern Biotechnology Associates, Inc., Brimingham, AL) for 1 hour at room temperature. Fluorescence was measured on a Cytofluor II multiplate fluorometer (Perceptive Biosystems, Framingham, MA) at an excitation wavelength of 485 nm and emission wavelength of 530 nm.

For flow cytometry, cells were detached from 60-mm culture plates with 0.5% EDTA solution containing matrix metalloproteinase inhibitor (KB8301, 10 μg/ml; Pharmingen) and incubated for 1 hour at room temperature with anti-FasL mAb (NOK1, 8 μg/ml) or isotype-matched mouse nonimmune IgG (Dako Cytomation Japan, Tokyo, Japan) in blocking buffer. Cells were then washed 3 times with PBS and stained with FITC-conjugated anti-mouse IgG antibody for 1 hour at room temperature. FITC-labeled cells were detected using a flow cytometer (FACS) on an FL-1 channel.

Amounts of soluble Fas L in samples of the supernatant from the 96-well plate cultures of HUVECs were analyzed by using a soluble FasL enzyme-linked immunosorbent assay (ELISA) kit (R&D systems Inc., Minneapolis, MN) according to manufacturer's instructions.

### Cytotoxicity assay

The ability of FasL on endothelial cells to induce apoptosis in Fas-positive target cells was assessed by co-incubating neutrophils with HUVECs. HUVECs were grown to confluence in 96-well plates and exposed to 100 μM H_2_O_2 _for 16 hours. The plates were then washed 3 times with PBS, and neutrophils (10^5 ^cells) suspended in EBM-2 medium lacking growth factors were added to HUVECs. The plates were centrifuged at 200 × *g *for 2 minutes, and after incubation at 37°C for 16 hours, non-adherent cells were collected and attached to glass slides by cytocentrifugation. The slides were fixed with a cold mixture of 60% acetone and 2% glutaraldehyde, reacted with a solution consisting of 250 μg/ml 3,3'-diaminobenzidine (Dako Cytomation) and 0.01% H_2_O_2 _for 10 minutes at room temperature, and stained with a Giemsa solution. Apoptotic neutrophils were identified as cells exhibiting nuclear pyknosis or chromatin condensation [12] together with brown-colored cytoplasmic staining when examined by oil immersion microscopy. Three hundred cells were scored in each experiment to determine the percentage of apoptotic neutrophils cells.

### In vitro culture of rat thoracic aortas

The Animal Care and Use Committee of Tokyo Women's Medical University approved the animal protocol. Thoracic aortas were obtained from male SD rats (Sankyo Labo Service Co., Tokyo, Japan) weighing 250 g, and they were cultured in the presence or absence of 100 μM H_2_O_2 _for 16 hours in EBM-2 medium lacking growth factors. The thoracic aortas were then fixed in 3% paraformaldehyde, and paraffin-embedded specimens were cut into 3-μm sections. Sections were deparaffinized, digested with a pepsin solution (Research Genetics, Huntsville, AL) for 5 minutes at 40°C, and exposed to 3% H_2_O_2 _for 20 minutes to inhibit endogenous peroxidase. After blocking with 3% bovine serum albumin and 2% goat serum, sections were incubated with polyclonal rabbit anti-FasL antibody (Wako Pure Chemical Industries, Ltd., Osaka, Japan) or non-immune rabbit IgG (Dako Cytomation) for 1 hour at room temperature. The primary antibody was reacted with biotin-conjugated anti-rabbit IgG antibody and streptavidin-horseradish peroxidase (Research Genetics). Immunoreactants were visualized with a substrate solution of 3-amino-9-ethylcarbazole (Dako Cytomation) and nuclear counterstaining with a hematoxylin solution.

### Statistics

All data are reported as means ± SEM. The mean values were compared by one-way analysis of variance (ANOVA) with Fisher's protected least significance difference (PLSD) *post hoc *analysis and the unpaired *t *test. A *p *value < 0.05 was considered significant.

## Results

### Oxidative stress increases FasL expression by HUVECs

**Figure 1:**
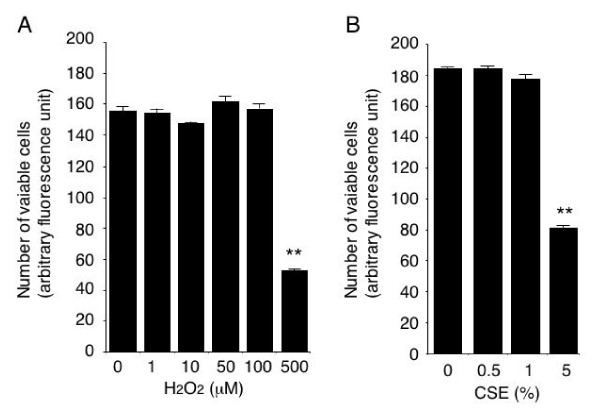
**Cell viability after exposure to H_2_O_2 _and CSE**. HUVECs were incubated for 16 hours in 96-well plates with or without 1–500 μM H_2_O_2 _or 0.5–5% CSE. Non-adherent cells were removed, and the number of adherent cells was counted by propidium iodide staining and spectrofluorimetry (13). **p < 0.01 vs cells not exposed to H_2_O_2 _or CSE.

**Figure 2:**
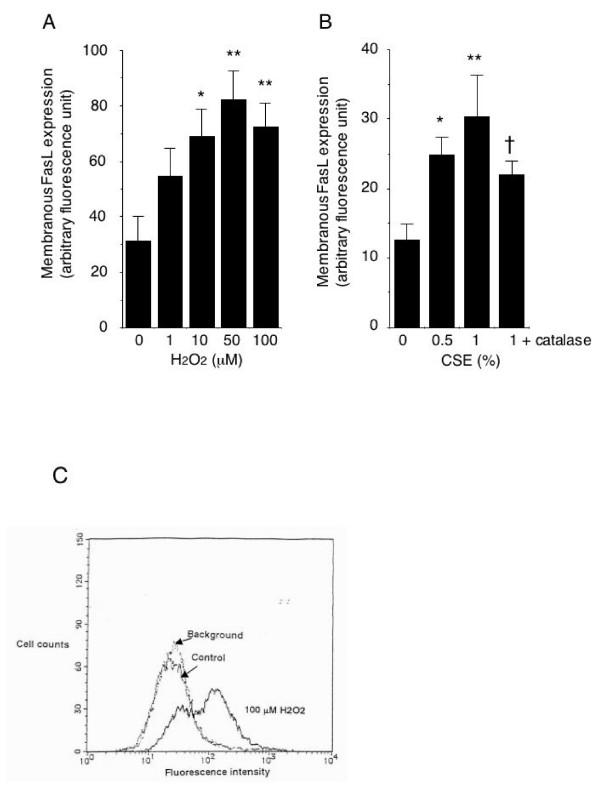
**Membrane FasL expression on HUVECs**. HUVECs were incubated for 16 hours with or without 1–100 μM H_2_O_2 _or 0.5% and 0.5 and 1% CSE, in the presence or absence of 500 U/ml catalase. Membrane FasL expression on HUVECs analyzed by cell-monolayer-based spectrofluorimetry (*A *and *B*) and flow cytometry (*C*). (*A *and *B*) Data are reported as the means ± SEM of the values obtained from 5 experiments. *p < 0.05, **p < 0.01 vs. cells not exposed to H_2_O_2 _or CSE. †p < 0.05 vs cells exposed to 1% CSE only. (*C*) Data are representative of 3 separate experiments. *Background*; fluorescence levels generated by replacement of anti-FasL antibody with nonimmune IgG.

Since as shown in Figure 1, exposure to H_2_O_2 _at concentrations of 1–100 μM and to CSE at concentrations of 0.5% and 1% did not affect HUVEC viability, we used these concentrations of H_2_O_2 _and CSE to assess their effect on FasL expression. The results showed that exposure of HUVECs to H_2_O_2 _dose-dependently increased the level of membrane FasL expression as assessed by cell-monolayer-based spectrofluorimetry (Figure 2A), and flow cytometry also showed an increased level of membrane FasL expression on HUVECs after H_2_O_2 _exposure (Figure 2C). Since cigarette smoke is an important source of oxidants that may affect endothelial function, we investigated whether CSE enhances membrane FasL expression by HUVECs. As shown in Figure 2B, HUVEC exposure to 0.5% and 1% CSE increased the level of membranous FasL expression. The increase was significantly inhibited in the presence of catalase, which catalyzes H_2_O_2_, suggesting that CSE induces FasL expression at least in part by oxidative mechanisms.

**Figure 3:**
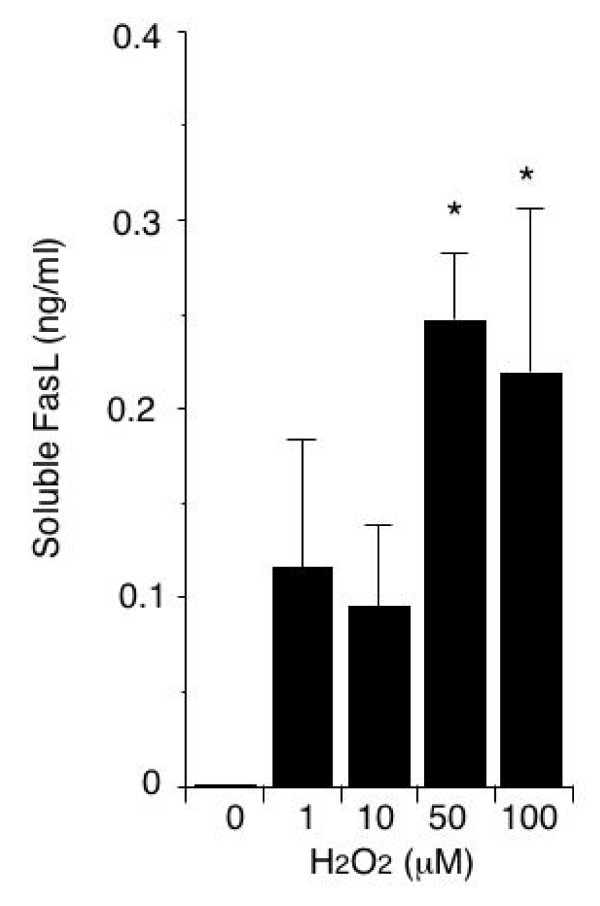
**Soluble FasL release by HUVECs**. Supernatant samples were obtained from cultures of HUVECs incubated for 16 hours with or without 1–100 μM H_2_O_2_. The amount of soluble FasL in the samples was determined by ELISA. Data are reported as the means ± SEM of values obtained from 5 experiments. *p < 0.05 vs. cells not exposed to H_2_O_2_.

Since membrane FasL is converted to a soluble form, we measured the amount of soluble FasL released by HUVECs exposed to H_2_O_2_. As shown in Figure 3, HUVEC exposure to H_2_O_2 _increased the amount of soluble FasL in the culture medium.

### Oxidative-stress-induced FasL expression by HUVECs induces neutrophil apoptosis

**Figure 4:**
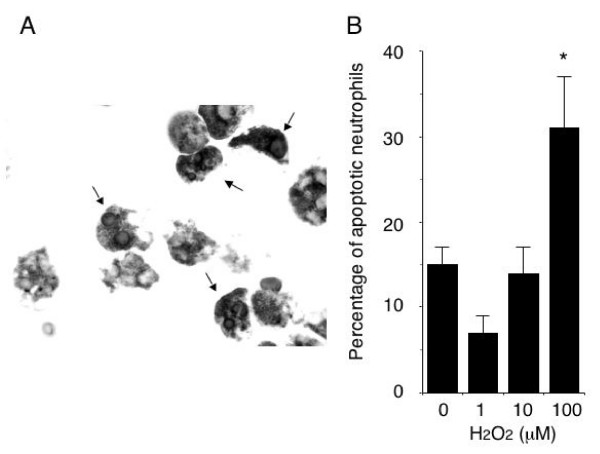
**Cytotoxicity toward neutrophils of HUVECs exposed to H_2_O_2_**. (*A*) A representative photomicrograph of neutrophils cultured for 16 hours with 100 μM H_2_O_2_-pretreated HUVECs and stained with diaminobenzidine and Giemsa solutions. *Arrows *point to apoptotic neutrophils with condensed and/or fragmented nuclei and cytoplasm positively stained with diaminobenzidine (brown in the original photograph). Original magnification, × 1000. (*B*) The number of apoptotic neutrophils co-cultured with HUVECs exposed to or not exposed to H_2_O_2_. Data are reported as the means ± SEM of values obtained from 5 experiments. *p < 0.05 vs. cells not exposed to H_2_O_2_.

To determine the functional capacity of HUVECs to induce apoptosis, we quantified Fas-positive neutrophils [14] co-cultured with HUVECs after exposure to H_2_O_2_. As shown in Figure 4, exposure of HUVECs to 100 μM H_2_O_2 _significantly promoted neutrophil apoptosis, suggesting that oxidative stress renders HUVECs cytotoxic to neutrophils.

### Oxidative stress increases FasL expression on the endothelium of the thoracic aorta

**Figure 5:**
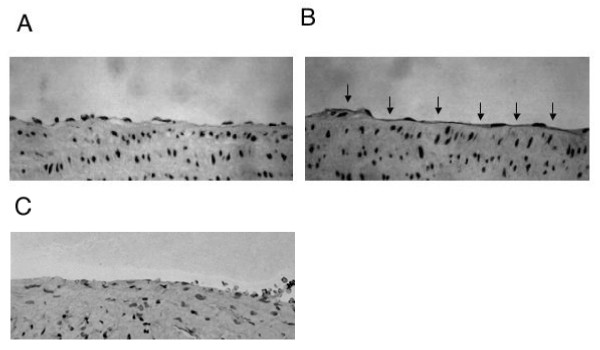
**Immunohistochemistry of thoracic aortas**. Thoracic aortas were excised from rats and exposed to (*B*, *C*) or not exposed to (*A*) 100 μM H_2_O_2 _in vitro for 16 hours. Tissue sections were immunostained with rabbit polyclonal anti-FasL antibody, and immunoreactants were visualized with a substrate solution of 3-amino-9-ethylcarbazole. Cell nuclei were counterstained with a hemtoxylin solution. Exposure of thoracic aortas to 100 μM H_2_O_2 _increased expression of FasL on the aortic endothelium (*B*). *Arrows *point to positive FasL staining (red color in the original photomicrograph). Replacement of anti-FasL antibody with non-immune rabbit IgG resulted in negative staining (*C*). Photographs are representative of immunostaining of three thoracic aorta specimens Original magnification, 200×.

The results obtained thus far had suggested that oxidative stress enhanced FasL expression in a HUVEC monolayer. We then investigated whether oxidative stress increases FasL expression on the endothelium of vascular tissues cultured in vitro. Since arteries have been shown to express low levels of FasL [4], we obtained thoracic aortas from rats and incubated them with or without 100 μM H_2_O_2_. Very weak immunoreactivity against anti-FasL was observed on the endothelium of thoracic aortas cultured with medium alone (Figure 5A), whereas culturing the thoracic aortas with H_2_O_2 _considerably enhanced endothelial immunoreactivity against anti-FasL (Figure 5B), suggesting that oxidative stress increases FasL expression on the endothelium of arteries.

## Discussion

The results of the present study showed that oxidative stress induced by nontoxic concentrations of H_2_O_2 _and cigarette smoke induces FasL expression on endothelial cells. Increased exposure of the endothelium to oxidants is involved in many pathological states, such as inflammation, ischemia/reperfusion, and atherosclerosis [11]. H_2_O_2 _is one of the most important oxidants derived from leukocytes and endothelial cells. It exerts a toxic effect on susceptible cells at high concentrations, but alters cell functions at low concentrations by modulating signal transduction pathways in certain cells, including endothelial cells [11]. Cigarette smoke is an important source of oxidants, including H_2_O_2_, and is thought to be a significant risk factor for chronic endothelial damage leading to atherosclerosis [15]. Our results suggest that low oxidant levels induce over-expression of FasL on the endothelium, which prevents inflammatory cell infiltration in tissue. This notion is consistent with our results showing that H_2_O_2_-exposed endothelial cells have increased ability to induce apoptosis in neutrophils bearing the Fas receptor [14]. Thus, FasL expression induced by oxidative stress may function to control inflammatory responses by reducing leukocyte extravasation.

Our results corroborate those of previous studies examining the effect of oxidative stress on membrane FasL expression in different types of cells, including T-lymphocytes [8], microglial cells [9], and intestinal epithelial cells [10]. However, the mechanism of FasL expression induced by oxidative stress is unclear. A previous report documented an association between oxidative stress-induced FasL expression and NF-κB activation, although the functional role of NF-κB has not been fully demonstrated [9,16]. Another possible mechanism of oxidative stress-induced FasL expression is tyrosine phosphorylation of signaling molecules, because tyrosine kinase-dependent signals, such as p38 mitogen-activated protein kinase and Jun-N-terminal kinase, have been implicated in FasL expression [17]. This possibility is supported by our preliminary experiments showing that H_2_O_2_-induced FasL expression in endothelial cells is enhanced by vanadate, a protein tyrosine kinase inhibitor, but reduced by genistein, a tyrosine kinase inhibitor (unpublished data).

Our results show that exposing HUVECs to H_2_O_2 _also increases the production of soluble FasL. Although the conversion of membranous FasL to its soluble form reduces its pro-apoptotic activity [18], soluble Fas ligand retains its capacity to interact with Fas, and serum concentrations of soluble FasL are related to the development of vascular diseases. For example, a recent study has shown that patients with familial combined hyperlipidemia or carotid atherosclerosis have decreased serum FasL levels, suggesting endothelial dysfunction that may allow leukocyte infiltration of the vessel wall and lead to atherosclerotic plaque formation [19]. In this context, the oxidative stress-induced increase in endothelial soluble FasL production observed in this study may contribute to maintaining homeostasis of the systemic vasculature.

Oxidative stress has been shown to alter various aspects of endothelial functions [11], for example, by increasing endothelial adhesiveness to neutrophils via signaling pathways dependent on activation of protein kinase C [20], production of platelet-activating factor [21], and expression of intracellular adhesion molecule-1 [22]. Thus, oxidative stress is generally thought to promote endothelial injury by enhancing leukocyte adhesion to the endothelium. In this sense, the endothelial FasL expression in response to oxidative stress demonstrated in this study may play a critical role in preventing endothelial injury by inducing apoptosis of adhering leukocytes.

We found that the addition of catalase only reduced CSE-induced FasL expression by HUVECs (Figure 2). Thus, an oxidative mechanism mediated by species other than H_2_O_2 _or non-oxidative mechanisms may also be involved in the increase in FasL expression by HUVECs after CSE exposure [23]. Alternatively, CSE may have inhibited catalase activity [24].

## Conclusion

The results of our study show that oxidative stress induced by nontoxic concentrations of H_2_O_2 _and CSE increase FasL expression in endothelial cells. This effect may contribute to reductions in leukocyte-mediated inflammation and tissue damage.

## Competing interests

The author(s) declare that they have no competing interests.

## Authors' contributions

SM and KA carried out all of the experiments reported in this manuscript. NA participated design of the study.
